# An Evidence-Based Nurse-Led Intervention to Reduce Diabetes Distress Among Adults With Type 1 Diabetes and Diabetes Distress (REDUCE): Development of a Complex Intervention Using Qualitative Methods Informed by the Medical Research Council Framework

**DOI:** 10.2196/58658

**Published:** 2024-10-18

**Authors:** Vibeke Stenov, Bryan Cleal, Ingrid Willaing, Jette Normann Christensen, Christian Gaden Jensen, Julie Drotner Mouritsen, Mette Due-Christensen

**Affiliations:** 1 Department of Prevention, Health Promotion and Community Care Steno Diabetes Center Copenhagen-Copenhagen University Hospital Herlev Denmark; 2 Section of Health Services Research Department of Public Health Copenhagen University Copenhagen Denmark; 3 Foundation for Mental Health and Cognitive Neuroscience Research Unit Aarhus University Aarhus Denmark; 4 Steno Diabetes Center Odense Odense University Hospital Odense Denmark

**Keywords:** diabetes mellitus, mental health, diabetes distress, qualitative research, co-design study, complex intervention, Medical Research Council framework

## Abstract

**Background:**

Diabetes distress refers to the negative emotional reaction to living with the demands of diabetes; it occurs in >40% of adults with type 1 diabetes (T1D). However, no interventions to reduce diabetes distress are specifically designed to be an integral part of diabetes care.

**Objective:**

This study aims to modify and adapt existing evidence-based methods into a nurse-led group intervention to reduce diabetes distress among adults with T1D and moderate to severe diabetes distress.

**Methods:**

The overall framework of this study was informed by the initial phase of the Medical Research Council’s complex intervention framework that focused on undertaking intervention identification and development to guide the adaptation of the intervention. This study took place at 2 specialized diabetes centers in Denmark from November 2019 to June 2021. A total of 36 adults with T1D participated in 10 parallel workshops. A total of 12 diabetes-specialized nurses were interviewed and participated in 1 cocreation workshop; 12 multidisciplinary specialists, including psychologists, educational specialists, and researchers, participated in 4 cocreation workshops and 14 feedback meetings. Data were analyzed by applying a deductive analytic approach.

**Results:**

The intervention included 5 biweekly 2.5-hour small group sessions involving adults with T1D and diabetes distress. Guided by a detailed step-by-step manual, the intervention was delivered by 2 trained diabetes specialist nurses. The intervention material included visual conversation tools covering seven diabetes-specific sources derived from the 28-item Type 1 Diabetes Distress Scale for measuring diabetes distress: (1) powerlessness, (2) self-management, (3) fear of hypoglycemia, (4) food and eating, (5) friends and family, (6) negative social perception, and (7) physician distress. The tools are designed to kick-start awareness and sharing of diabetes-specific challenges and strengths, individual reflections, as well as plenary and peer-to-peer discussions about strategies to manage diabetes distress, providing new perspectives on diabetes worries and strategies to overcome negative emotions. Diabetes specialist nurses expressed a need for a manual with descriptions of methods and detailed guidelines for using the tools. To deliver the intervention, nurses need increased knowledge about diabetes distress, how to support diabetes distress reduction, and training and supervision to improve skills.

**Conclusions:**

This co-design study describes the adaptation of a complex intervention with a strong evidence base, including detailed reporting of the theoretical underpinnings and core mechanisms.

## Introduction

### Background

It is estimated that >40% of adults with type 1 diabetes (T1D) experience diabetes distress, defined as the emotional concerns of people living with and managing diabetes [1,2], in the course of time. Diabetes distress is conceptually distinct from depression; however, diabetes distress tends to be chronic rather than episodic compared with depression [3]. Furthermore, when compared with depression, diabetes distress is independently and more strongly associated with diabetes self-management metrics and glycemic levels outside the recommended range in both cross-sectional and longitudinal studies [1,4-7]. It has been suggested that people with diabetes distress may be inaccurately diagnosed with depression, diverting the attention of health care professionals from diabetes distress, leading to ineffective treatment [8].

Given the high prevalence of diabetes distress, various international diabetes guidelines highlight the importance of integrating systematic screening for providing interventions to reduce diabetes distress [9]. Despite these recommendations, identifying and reducing diabetes distress is currently not part of routine diabetes care [9]. In general, health care professionals spend minimal time discussing the psychosocial aspects of diabetes during consultations [10,11]. This might be partly due to structural barriers, such as limited time for consultations, but may also involve a lack of training and tools to address psychosocial aspects of diabetes within clinical care [10,12,13].

A recommended approach to detecting diabetes distress is to use diabetes-specific questionnaires [9,14]. The Type 1 Diabetes Distress Scale is a reliable and valid 28-item self-report instrument developed to uncover the multidimensionality of diabetes distress. This scale identifies specific sources of diabetes distress for adults with T1D and provides an index of overall diabetes-related distress. It is assigned a total score and seven factor-derived, specific subscales related to diabetes distress: (1) powerlessness, (2) management distress, (3) hypoglycemia distress, (4) eating distress, (5) friends and family distress, (6) negative social perceptions, and (7) physician distress [15,16]. These distinct sources of diabetes distress among adults with T1D can be used to guide conversations about psychosocial aspects of living with diabetes [16,17].

Reviews of interventions to reduce diabetes distress among adults with diabetes indicate that diabetes distress is responsive to interventions and observed effects persist over time with minimal relapse [7]. A systematic review and meta-analysis by Schmidt et al [7] demonstrated that elevated symptoms of diabetes distress can be alleviated by tailored psychological interventions. A meta-analysis of 41 randomized controlled trials conducted by Sturt et al [18] found that the most effective interventions to reduce diabetes distress specifically targeted emotional aspects rather than focusing on behavior change or education.

One of the few efficacy trials targeting diabetes distress is the Randomized Controlled Trial to Reduce Diabetes Distress Among Adults With Type 1 Diabetes (T1-REDEEM). T1-REDEEM showed dramatic reductions in diabetes distress after 3 months post the intervention. This effect was sustained at 9 months with further modest improvements [19]. The T1-REDEEM study was primarily conducted by trained psychologists in controlled settings; however, the availability of psychologists in real-world clinical settings is generally limited [19]. Furthermore, research shows that clinical health care professionals generally recognize the importance of addressing and supporting the psychosocial needs of people with diabetes [20]. In line with this, many adults with diabetes distress want to talk about their emotional well-being with their health care professionals [13].

As diabetes distress is common and closely linked to diabetes management, existing evidence suggests that interventions should be implemented and integrated within the context of routine diabetes care. Diabetes specialist nurses (DSNs), who play a substantial role in delivering diabetes care as part of the multidisciplinary health care team, could be a vital resource in providing support for reducing diabetes distress if they receive specialized training [21]. Given the fact that many diabetes specialist settings worldwide lack access to psychologists, the development of materials and programs to inform and assist DSNs in delivering interventions to reduce diabetes distress could potentially expand the reach of diabetes distress support to a broader population of adults with T1D. However, it remains unclear how such reduction strategies could be organized to make it easier for DSNs to provide this support systematically in real-world, clinical diabetes-care settings.

### Objective

This study aimed to modify and adapt existing, evidence-based methods into a nurse-led group intervention in clinical practice to reduce diabetes distress among adults with T1D and moderate to severe diabetes distress.

## Methods

### Study Design

This study was undertaken in the first phase of the revised complex intervention framework of the Medical Research Council (MRC), intervention identification and development, before testing and evaluating the intervention [22,23]. According to the MRC framework, the identification and development phase involves the inclusion of existing interventions and methods; the best available evidence-based research with the potential for adaptation to a new population in a new context is selected.

### Intervention Identification: Existing Methods for Modeling the Intervention

Initially, existing evidence on the most effective methods to reduce diabetes distress in adults with T1D was identified to develop an intervention model. Exploration of effective delivery methods was also done by DSNs.

The Evidence-Based Nurse-Led Intervention to Reduce Diabetes Distress Among Adults With T1D and Diabetes Distress (REDUCE) was mainly inspired by the T1-REDEEM study, specifically the emotion-focused OnTrack intervention conducted by Fisher et al [19]. This included exploring necessary skills related to delivering the intervention. In addition, the intervention was inspired by the evidence-based participatory health education concept Next Education, developed by Engelund et al [24] at Steno Diabetes Center Copenhagen [24,25]. Finally, the development of REDUCE was inspired by components from the evidence-based Open and Calm model developed in Denmark, specifically for public stress-reduction initiatives [26].

T1-REDEEM identified emotion regulation and peer support as effective mechanisms for reducing diabetes distress. It incorporates techniques such as emotional management strategies, which includes labeling emotions, maintaining feelings in perspective, and separating emotions from appraisals of self-worth [19], drawing from empowerment-based communication [27] and motivational interviewing [28].

Emotion regulation involves influencing the experience, expression, and effects of emotions and is linked to diabetes distress through factors such as self-awareness, mindfulness, acceptance, and the ability to act on a plan [19].

Peer-support elements enable sharing of experiences among peers (adults with T1D) and tap into social learning theory and social comparison theory by normalizing emotions, alleviating isolation, and promoting a sense of relatedness and hope [29-31]. Peer support also enhances emotion management skills by reducing overreactions and increasing awareness of self-blame tendencies through sharing alternative ways of reacting to stressful diabetes experiences [19].

Reviews by Fisher et al [14,21] have provided practical frameworks to translate the theoretical concepts about addressing diabetes distress into practical strategies used in clinical settings. These frameworks were based on the self-determination theory [32] and informed the development of a conceptual framework guiding DSNs in delivering the distress-reducing intervention. Overall, the framework emphasizes a shift from a traditional information deliverer and problem-solver role to an active explorer role, promoting person-centered conversations to support the emotional aspects of living with diabetes. Professional skills align with self-determination theory and include motivational interviewing strategies and empowerment-based communication, involving abilities such as acknowledging feelings, summarizing, normalizing, offering new perspectives, and developing follow-up plans [14].

Next Education, a health education concept, uses social learning theory and empowerment to enhance self-management skills in individuals with chronic conditions [25]. Using dialogue tools, such as images, peer quotes, and reflective questions, it facilitates tailored patient education using a health education model with 4 professional roles—the embracer, the facilitator, the translator, and the initiator. The embracer fosters a supportive environment; the facilitator sparks dialogue; the translator imparts person-centered knowledge; the initiator motivates the participants to enact positive life changes [24,33].

The Open and Calm model is a health psychological integrative common-factors model developed for public stress-reduction initiatives. It has shown substantial effects on stress compared with regular care [26]. Similarly, the Open and Calm model substantially improved the quality of life compared with regular care when applied in the public health sector for patients with inflammatory diseases [34]. The practical implementation of the model has been continually refined since 2014. The model has been recommended as an inspirational model for public stress-reduction programs by the Danish Medicine and Health Authorities [35].

### Intervention Development: Adapting and Modeling Methods to Shape the Intervention

#### Overview

The adapting and modeling process was inspired by the core elements within each research phase in the MRC framework; for example, developing and refining program theory, engaging stakeholders, and refining the intervention [23]. Aligned with the MRC framework, this involved refining or making changes to a preliminary set of prototypes with relevant stakeholders in workshops and interviews. This process has the potential of developing or identifying an intervention that is likely to have positive impacts in practice [19]. Thus, existing available methods and strategies, including the theoretically hypothesized mechanisms of effect for reducing diabetes distress, were adapted to shape the REDUCE intervention.

The study was conducted at Steno Diabetes Center Copenhagen and Steno Diabetes Center Zealand. Steno Diabetes Center Copenhagen is a specialist diabetes clinic providing diabetes care services to approximately 6000 adults with T1D and who live in the Capital Region of Denmark; Steno Diabetes Center Zealand has approximately 3200 adults with T1D based in Region Zealand. Adults with T1D were conveniently sampled through patient and public involvement groups at Steno Diabetes Center Copenhagen or Steno Diabetes Center Zealand and via information screens in the clinics and on the hospitals’ websites. Interested participants received additional information about the study via email and a telephone call. DSNs from Steno Diabetes Center Copenhagen were invited to participate via email.

Eligibility criteria for inclusion of people with T1D were ≥18 years of age at the start of the study and being able to understand and read information in Danish. Exclusion criteria were serious mental illness (eg, major depression and schizophrenia) or inability to speak and read Danish. Eligibility criteria for health care professionals included having >1 year of experience in providing specialized diabetes care for adults with T1D.

#### Workshops With Adults Having T1D and Experts Meetings: Adapting Methods

A total of 10 workshops with adults having T1D, involving 4 to 10 participants in each workshop (n=36), were conducted in person at the 2 participating diabetes clinics. The workshops were facilitated by members of the research team consisting of experienced DSNs, psychologists, and scientists. Participants were informed about the study objective and the reasons for conducting the study. Workshops with adults with T1D lasted for 3 hours, with breaks for food and refreshments. During the workshops, ideas and prototypes of existing evidence-based methods were presented, tested, and commented on by participants to explore their potential utility in initiating or strengthening dialogue and discussion about the 7 dimensions of diabetes distress and possibly other sources of diabetes distress. The adaptations of the prototypes based on findings from the previous workshop were presented for adults with T1D in the following workshop. This was done to give participants the opportunity to provide comments and feedback on the findings.

#### Workshop and Interviews With Health Care Professionals: Nurses’ Crucial Skills Set

A total of 12 DSNs participated in a 2-hour workshop in person; moreover, 1 individual semistructured face-to-face interview was conducted with each nurse until data saturation was reached. This was done to explore the skills needed to deliver the intervention. An interview guide was developed based on the findings from previous research and was discussed among the research team. Interviews with DSN lasted between 45 and 90 minutes. In addition, 14 feedback meetings with psychologists, educational specialists, and researchers with expertise in user-driven innovation were conducted. These meetings were either face-to-face or web-based and were conducted to refine the prototype content. The feedback was used to make adjustments regarding how wordings, questions, and illustrations could be optimized in the visual conversation tools as well as to optimize the overall REDUCE program. A summary of data types and collection points is shown in Table 1.

**Table 1:** Summary of study data types and collection points.

Data collection point			Objective		Participants		Data types
**Modeling evidence-based methods into a new context (adults with type 1 diabetes)**							
	Workshops for adults with diabetes (n=10)	To generate, develop, and test ideas for usability, relevance, and design		Adults with type 1 diabetes (n=36)		Transcription of audio-recorded group discussions	
	Workshops for health care professionals, SDCC^a^ (n=3)	To generate, develop, and test ideas for usability, relevance, and design		Diabetes educators (n=3; 1 physician and 2 nurses)		Transcription of audio-recorded group discussions	
	Web-based workshop (n=1)	To generate, develop, and test ideas for usability, relevance, and design		Multidisciplinary researchers specializing in user-driven innovation (n=5)		Preliminary ideas on sticky notes and field notes	
	Feedback meetings (n=14)	To present and obtain structured feedback on preliminary prototypes, including a step-by-step training manual explaining how nurses can use each tool		Psychologists (n=4)		Field notes	
**Exploring necessary professional skills to deliver the intervention (diabetes specialist nurses)**							
	Workshop (n=1), SDCC	To identify preferences and needs related to facilitating diabetes distress support methods		Nurses (n=12)		Field notes and transcription of audio-recorded group discussions	
	Individual interviews with diabetes specialist nurses, SDCC	To explore—enablers and barriers to facilitating diabetes distress support methods		Nurses (n=12)		Transcription of audio-recorded interviews	

^a^SDCC: Steno Diabetes Center Copenhagen.

### Data Collection

Data consisted of transcribed audio recordings of interviews, workshop discussions, and field notes from interactive workshops and meetings as well as sticky notes and flip charts from feedback meetings. Data were collected from November 2019 to June 2021; data collection was primarily conducted by the first author (VS). However, this study was undertaken during the COVID-19 pandemic. As a result, the study, which was advertised via on-screen information, primarily enlisted adults with T1D through a diabetes panel affiliated with both Steno Diabetes Center Copenhagen and Steno Diabetes Center Zealand.

### Data Analysis

All workshops and interviews were audio-recorded and transcribed verbatim. Workshop and interview transcripts were sorted and analyzed using Word (Microsoft Corp). Data from interviews, workshops, and feedback meetings were analyzed by applying a deductive analytic approach to systematic interpretation and categorization of data. Initially, 3 coders performed a broad coding of the data by reading all transcripts. The initial coding patterns and themes were inspired by established evidence-based theories, concepts, and methods chosen in the intervention identification phase before developing and adapting the intervention. These methods comprised the self-determination theory, including motivational interviewing strategies and empowerment-based communication as used in the T1-REDEEM study. Finally, codes were sorted into potential themes augmented by quotations from the raw data underpinning the findings, which were discussed within the research group to reach consensus.

### Ethical Considerations

The study was approved by the Danish Data Protection Agency (approval number P-2020-90). The Danish Committee on Health Research Ethics was informed about the project according to the Danish Act on Research Ethics Review of Health Research Projects (H-22038926). Participation was voluntary, and written informed consent was obtained from participants and all data were anonymised.

## Results

### Overview

In total, 36 adults with T1D (Table 2) accepted the invitation to participate and took part in the study. A total of 12 DSNs and 12 experts, including psychologists, diabetes educators, and researchers participated in the study. The median age of adults with T1D was 58 (IQR 50-61.2) years and 23 (64%) were female. The median diabetes duration was 33 (IQR 2-46) years. For glucose monitoring, 29 (81%) used a sensor. In total, 19 (53%) adults used an insulin pen, and 17 (47%) adults used an insulin pump for insulin delivery.

**Table 2:** Characteristics of people with type 1 diabetes participating in the study (n=36).

Characteristic		Participants
Female, n (%)		23 (64)
Age (y), median (IQR)		58 (50-61.2)
**Education, n (%)**		
	Primary school	1 (3)
	Upper secondary school	2 (5)
	Vocational school	8 (22)
	Short higher education	6 (17)
	Medium higher education	9 (25)
	Long higher education	10 (28)
**Cohabitation, n (%)**		
	Lives alone	9 (25)
	Lives with partner or children	27 (75)
Diabetes duration, median (IQR)		33 (20.3-46)
**Diabetes complications, n (%)**		
	Retinopathy	14 (40)
	Nephropathy	2 (6)
	Neuropathy	9 (25)
	Foot ulcers	1 (3)
	Macrovascular	6 (17)
**Glucose monitoring, n (%)**		
	Strips	4 (11)
	Sensor	29 (81)
	Sensor and strips	3 (8)
**Insulin delivery method, n (%)**		
	Pump	17 (47)
	Pen	19 (53)

The adaptation and modeling process, drawing upon evidence-based interventions presented in the Methods section was subsequently refined through feedback from adults with T1D, health care professionals, and researchers. This resulted in the development of the REDUCE intervention. Participants’ content and delivery needs, including illustrative quotes, are described in Table 3. Content needs of the intervention were centered on 5 categories: (1) sharing experiences among peers, (2) gaining knowledge about diabetes distress, (3) labeling and verbalizing unrecognized worries, (4) developing strategies to manage diabetes distress, and (5) normalizing and putting emotions in perspective. Adults with T1D expressed the following needs regarding the delivery of the intervention: the facilitator should be able to (1) guide respectful and supportive group interactions with a clear structure and set boundaries for maintaining privacy and (2) enhance engagement and learning through visual conversation tools.

**Table 3:** Categorization of content and delivery needs, including illustrative quotes.

Categories and needs from participants			Illustrative quotes and excerpts from workshop discussions
**Content of the intervention**			
	Sharing experiences among peers	Workshop 1: “I believe that being able to talk about it can help alleviate diabetes-related distress. The opportunity for a dialogue about these things with others in the group, because with one’s family, you can’t always talk about this. We specifically discuss these things when we’re here with others who understand it. Hearing from others who talk about it allows you to relate to it.” Workshop 2: “There can be something really good about hearing from someone who thinks completely differently than oneself—it can create some perspective and thoughts that can be useful. Workshop 4: We need others with diabetes and to be part of a community.” Workshop 7: “Being 6 to 8 people and hearing from others. That also provides some answers.”	
	Gaining knowledge about diabetes distress	Workshop 8: “No one has ever asked if you have diabetes distress. I am speechless now as I sit here hearing about diabetes distress. All these points that come up (knowledge about diabetes distress), well, we have always had that, but no one has ever gone into it.” Workshop 8: “It’s an acknowledgement (gaining knowledge about diabetes distress), and that’s half of it. The fact that there’s a focus on it—and what can we do and how can we gain support.” Workshop 1: “It can be quite comforting to know that it’s quite common to be burdened by the illness. So, you don’t feel completely foolish because you can’t even handle it.” Workshop 4: “I thought it was such an ‘aha’ experience: I thought, oh, that’s it. It’s nice that words are put to it. Like the others, I can check all the boxes, but I’ve carried it a lot myself, so it’s nice because we don’t talk about it at the hospital. We talk a lot about numbers and blood sugar, but if you talk about diabetes distress, then you’re not so alone with it. It’s like a knot in the stomach that you carry around: It’s nice to be informed about it.”	
	Labeling and verbalizing unrecognized worries	Workshop 3: “You mirror yourself a lot in the statements. And the ability to elaborate on them makes them more personal.” Workshop 7: “It’s important and a very good starting point for putting words to something, and a good way for health care professionals to gain insight into what one participant is thinking, which is very crucial. It’s a way to give us a voice and express something, allowing for an open conversation.” Workshop 7: “It opens the lid and serves as a tool to uncover things that one can address, providing more focused support.” Workshop 8: “We have different perspectives, our individual ways of being, and approaches to illnesses—and there is ample space for that here with the conversation cards, and dialogue cards in a group. The cards offer various perspectives, covering a wide range of aspects.”	
	Developing strategies to manage diabetes distress	Workshop 1: “I think a lot about strategies, so how do you take that next step? One thing is recognition—yes, okay, check, check, check. And then what? How do I move forward from here? How can I improve a bit—just, you know, think about something in a different way? Get some inspiration for that. I feel like my thoughts are stuck on diabetes. How do you get out of it?”	
	Normalizing and putting emotions into perspectives	Workshop 1: “It brings a sense of peace to my mind. It’s nice to know that it (managing diabetes) is not easy and others are feeling the same way. You are not alone, and it gets normalized. To be reassured that you are doing it well enough. It’s an aha experience for me, a reassurance, to get some knowledge that informs us that others also feel the same way, then maybe it will be possible to put it in perspective with others with whom we compare ourselves.”	
**Delivery of the intervention**			
	Guiding respectful and supportive group interactions with a clear structure and set boundaries for maintaining privacy	Workshop 1: “I believe it would be helpful to have an introduction to some of the topics before people have to fill something out, as some are very private. It can be almost intimidating, for example, to talk extensively about one’s food, but discussing one’s food in this way is very private. However, it shouldn’t be wrapped up; it should be unfolded. But food is very intimate and tabooed—this forum is good for talking about it—because there are people who understand me. But we need to be aware that this belongs to something private, perhaps not even known by your closest family. It’s too much to be thrown into it without an introduction. A greater awareness that this was what we were supposed to do from the beginning—and an awareness that there’s nothing right or wrong. There should be no pressure if you don’t want to share it.”	
	Enhancing engagement and learning through visual conversation tools	Workshop 8: “Some people respond better to things that engage other senses. To sharpen focus and concentration, and that, I think, is important.” Workshop 1: “The descriptions have to be conveyed in a visual way, then it is easier to translate into everyday life.”	

REDUCE consists of a toolbox that has 14 visual conversation tools and a manual. Each tool incorporates visual aids, illustrations, and ongoing hands-on activities presented in easy-to-understand language with concrete examples. The manual includes a detailed step-by-step guide that supports DSNs to use the tools appropriately [36]. REDUCE was feasibility tested in the format of 5 biweekly 2.5-hour small group sessions involving 8 to 12 people with T1D and moderate to severe diabetes distress. It was delivered by 2 specifically trained DSNs [37]. The manual for REDUCE and the guidelines for using the visual conversation tools are available from the authors on request; however, information about the tools are currently available only in Danish language.

The adaptation process of REDUCE is presented as follows: (1) the development of visual conversation tools and (2) the development of the necessary skill set, among nurses, which are required to use the visual conversation tools effectively.

### Development of Visual Conversation Tools

**Figure 1:**
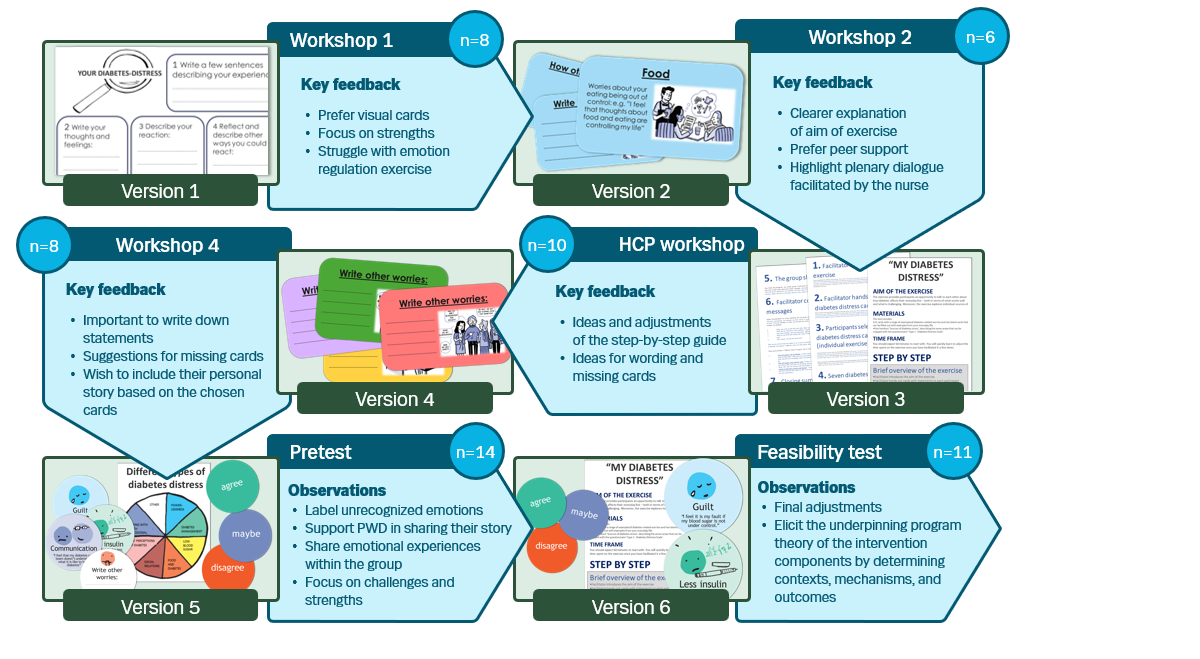
The iteration process of the prototype tool My Diabetes Distress from idea to final conversation tool. PWD: people with diabetes.

This section contains (1) an example of the iterations that led to a final tool (illustrated in Figure 1), (2) descriptions of the shared mechanisms and strategies embedded within most of the visual conversation tools, and (3) an overview of the visual conversation tools (Multimedia Appendix 1), including descriptions of the unique mechanisms for each of the tools.

The visual conversation tools underwent 5 to 15 adaptive iterations to ensure the acceptability of the intervention in clinical care. Figure 1 exemplifies the iterative process of the visual conversation tool My Diabetes Distress from its conceptualization to the final prototype that is ready for pilot testing.

### Overall Mechanisms and Strategies Embedded in Visual Conversation Tools

In the following sections, the shared mechanisms and facilitation techniques incorporated in most of the visual conversation tools are outlined.

#### Express and Release Emotions to Promote Clarity and Self-Awareness

Integrating illustrations, statements, or reflective questions in visual conversation tools may act as a catalyst for people with diabetes, facilitating the articulation and sharing of often unspoken and unrecognized worries and frustrations with peers. Thus, participants are, for example, presented with a pile of cards, with each card containing various statements representing different experiences and emotions related to the 7 different sources of diabetes distress. The participants are encouraged to initially read visual cards with statements and then sort and place them into 3 different piles: *agree*, *maybe,* and *disagree*. Once the quotes are sorted individually, the DSNs divide the participants into small groups and encourages them to share their choice of quotes and express the emotions associated with them. Subsequently, the peer discussion is summarized within the whole group to reinforce key insights and a sense of group cohesion.

Overall, this process allows participants to release pent-up emotions and provides them with greater emotional clarity, self-awareness, and a deeper understanding of their own reactions. Allowing participants to label and read their concerns in the form of statements made by others can make them appear more objective and less personal, empowering participants to deal with worries associated with diabetes. The tools may also equip participants with a novel vocabulary, which can facilitate the communication of often difficult emotions more comfortably. In addition, placing worries in the *disagree* pile allows participants to gain insights into what is less challenging for them, thereby enabling them to focus on their successes as well. Enhanced awareness of success can reduce self-blame and increase self-esteem and motivation, thereby helping them to continue working on what is difficult.

#### Normalize Challenges to Reduce Self-Blame and Isolation

The statements on visual tools support the articulation of common worries as well as foster normalization, validation, and acceptance of emotions. Framing and separating emotional challenges as external statements on cards rather than appraisals of self-worth can help separate challenges from identity. This separation helps prevent feelings of self-blame and feelings of shame and guilt, allowing a more objective and constructive approach. By sharing similar stories, participants recognize the normalcy of their feelings, which can promote a sense of relatedness and reduce isolation. The supportive group environment ensures that participants feel heard and understood, reinforcing a sense of belonging and alleviating diabetes-related loneliness.

To activate normalization, validation, and acceptance of emotions, dialogue cards entitled “Did you know...” are included. These cards describe evidence-based facts and knowledge about the prevalence, causes, symptoms, common responses, and potential consequences of diabetes distress. These cards also include information about the actual risk of serious complications or the impact of social relations and stigma in adults with T1D. Fact cards are handed out with a short description in an easy-to-understand language. To convey information, visual elements such as diagrams, charts, and images are also included in these cards. Participants are asked to read the cards and choose the most important or relevant cards for further discussion or elaboration within the group.

#### Develop Strategies to Manage Diabetes Distress

Nonjudgmental, reciprocal listening, and story-sharing among peers to reflect and discuss similarities and differences that arise from participants’ different perspectives can lead to the discovery of new insights and alternative strategies for managing diabetes distress. Reflective questions on the visual conversation tools can empower participants to take control of their concerns by identifying the root of the concerns and exploring concrete perspectives and practical steps to overcome specific situations. To develop strategies that help manage diabetes distress, the conversation tools incorporate elements that invite participants to reflect and share strategies to overcome challenges, discuss ways to restructure negative thoughts, and identify concrete and practical steps to overcome worries (tools: *worries about low blood glucose* and *food and eating*). In addition, exercise sheets with various reflective questions, with allotted space for participants to write their notes, are handed out to enhance the development of emotion-regulation skills (tools: *worries about my blood glucose* and *friends and family support*). Each question is introduced and expanded upon by the nurse, giving space for individual reflection before peer discussions. The questions address the following aspects: (1) specific events or circumstances contributing to or triggering emotional distress to identify the roots of worries, (2) identifying alternative perspectives that may exist in a specific situation, and (3) identifying concrete and practical steps to overcome worries.

#### Foster Hope to Increase Empowerment

The tools include questions aiming to elicit positive emotions and explore participants’ successes in overcoming diabetes-related challenges to counterbalance negative emotions. Fostering hope combats despair, cultivating a sense of optimism, enthusiasm, and empowerment. This approach may enhance self-efficacy and contribute to a positive outlook on managing diabetes. Lowering the intensity of negative emotions can support participants to view challenges with greater objectivity, which can empower the participants’ problem-solving skills to manage diabetes distress more effectively.

Focusing exclusively on negative emotions can generate despair or hopelessness. To avoid this, the tools also contain questions to elicit positive emotions such as optimism and enthusiasm. This can be done by exploring how participants have overcome their obstacles and succeeded in their lives with diabetes. Fostering hope counteracts negative feelings and can enhance self-efficacy and empowerment.

To elicit positive emotions, open-ended questions focusing on less-challenging events or emotions are embedded in tools. An example in this regard is an exercise that helps participants in sharing their diabetes story (tool: My diabetes story). Likewise, participants are asked to choose 2 cards containing statements that they have sorted into their *disagree* pile. This is done to focus on what currently works well for them or what they do not struggle with (tool: *my diabetes distress*, *worries about low blood glucose*, *food and eating*, and *family and friends*).

### Descriptions of All Visual Conversation Tools, Including Unique Mechanisms

Table 3 gives an overview of each of the tools, designed to target the 7 most common sources of diabetes distress as assessed by the Type 1 Diabetes Distress Scale [15]. Some of the visual conversation tools are mandatory to use, whereas others can be selected by the DSNs to align with the unique sources of diabetes distress among participants in the group. Three of the visual conversation tools set the overall framework of the intervention, and 11 tools aim to reduce diabetes distress.

### Necessary Skill Set, Required to Use Visual Conversation Tools Effectively, for Nurses

Facilitating group dynamics was perceived by nurses as the most challenging part of delivering the intervention: *“*The facilitator role is the most demanding compared with the others (skills). It is something that you need to practice*”* (Participant 5). The nurses suggested in interviews and the workshop that *“*it would require that we do some roleplaying beforehand where you train the material with your colleague.... It’s something that really requires thorough preparation, also mentally*”* (Participant 6). In addition, “there has to be some sort of supervision where you can discuss: what went well and how else could we have done it*?*” (Participant 9, workshop). “eg, to try it out a few times under supervision from one of the psychologists” (Participant 2).

As informed by Fisher et al [14], the required skill set to facilitate the intervention was identified in their practical strategies for addressing diabetes distress in clinical settings. This was supplemented by input from DSNs, educational specialists, psychologists, and researchers involved in the intervention design. The final skill set for DSNs to facilitate REDUCE is described in Textbox 1.

**Professional skills needed to facilitate the evidence-based nurse-led intervention to reduce diabetes distress among adults with type 1 diabetes and diabetes distress (REDUCE).**

**Establishing and maintaining an empathic, safe, and confidential group environment**

- Setting up group ground rules for respectful, nonjudgmental, open, supportive, and compassionate communication within the group
- Encouraging group participants to consider what they are comfortable sharing with the whole group to respect personal boundaries

**Providing clear and transparent communication**

- Clearly articulating the agenda for each of the meetings in collaboration with the group, including topics to be discussed and the allocated time.
- Introducing expectations of the group activities and the various steps of the visual conversation tools that encourage interaction and group dialogue
- Being prepared to adjust the agenda and time when important group discussions arise

**Demonstrating genuine interest and attention to support the group in expressing and sharing their emotions**

- Asking open-ended questions to open group discussions that prompt participants to share experiences
- Summarizing and repeating the content of participants’ statements and conclusions to reassure participants that they have been heard accurately
- Identifying and summarizing shared experiences among the group participants

**Normalizing and validating diabetes experiences and emotions**

- Expressing empathy by acknowledging and affirming that negative emotional experiences are expected under the circumstances
- Validating without fixing or offering advice
- Facilitating the sharing of similar emotions among peers
- Respecting the uniqueness and diversity of individual experiences
- Reminding participants that no emotions are inherently good or bad; it is how we respond to and manage them

**Encouraging participants to verbalize and label unarticulated emotions**

- Creating space for sharing experiences among peers
- Prompting writing by asking reflective questions

DSNs must have an empathic mindset and must establish a collaborative interactional style to use the tools effectively. This requires slowing down the pace of speech to allow silence for reflection and being comfortable with discussing challenging emotional topics. In addition, it is important to guide the conversation through curious and nonjudgmental open-ended questions and provide affirming and acknowledging statements to focus on the needs of the adults with T1D rather than the nurses’ needs.

## Discussion

### Principal Findings

This study undertook the initial phase of the MRC framework to identify, define, and develop the underlying theoretical considerations of a group-based diabetes distress reduction intervention (REDUCE) for transparent and comprehensive reporting of the intervention. Development of the intervention theory included initial identification of preexisting theoretical and empirical evidence to inform the preliminary content and delivery methods. The REDUCE intervention was designed primarily based on methods from the existing diabetes distress reduction large-scale efficacy trial T1-REDEEM [19]. However, efficacy trials are typically conducted in ideal and tightly controlled conditions. What REDUCE adds is the integration of the perspectives of adults with T1D, clinical health care professionals, and relevant experts within the field to further design, develop, and adjust the intervention into a real-world clinical setting. This led to the creation of a set of visual conversation tools that address the 7 sources of diabetes distress. These tools were designed to kick-start awareness and share diabetes-specific challenges and strengths, which included individual reflections as well as plenary and peer-to-peer discussions about strategies to manage diabetes distress. This process provides new perspectives on diabetes-specific worries and frustrations and strategies to overcome negative emotions.

The visual conversation tools are accompanied by a detailed manual offering in-depth explanations of methods and detailed step-by-step guidelines for using the REDUCE tools effectively by enabling DSNs to systematically and effectively support the reduction of diabetes distress in adults with T1D [21]. It can be challenging to develop such professional skills; training materials are not sufficient to establish the necessary skill set. Before delivering REDUCE, DSNs need to be trained in the intervention curriculum and relevant facilitation skills in a 1.5-day workshop. In addition, clinical supervision after each group session with REDUCE trainers in the implementation period needs to be accessible to build sufficient capability to deliver REDUCE and ensure effective implementation of the intervention in practice [23,38]. In addition, access to qualified professionals, such as psychologists, is recommended to ensure ongoing clinical supervision for nurses to enhance their skill sets if difficult situations arise.

This study aimed to include a detailed description of the theoretical underpinnings and core mechanisms of REDUCE. A study published in 2023 by Stenov et al [37] reported the effectiveness of REDUCE in a pilot study. The study showed overall statistically significant reductions in diabetes distress in the short term. Positive effects, such as improvement in the quality of life and empowerment of the affected individuals [37], accompanied. In comparison, the US-based, theory-based diabetes distress reduction intervention T1-REDEEM displays dramatic reductions in diabetes distress 3 months after the intervention. This effect was sustained 9 months after the intervention, with further modest improvements [19]. Future studies need to investigate the effectiveness of REDUCE in a longer period after the intervention, including outcome measures during 3-, 6-, and 9-month follow-ups after the intervention.

### Importance of Theory-Building in the Development of Complex Interventions

A recent rapid review conducted by Wallner et al [39] outlines that it is crucial to ground the development of nursing interventions in underlying theory. Within the MRC framework, it is essential not only to focus on defining the anticipated outcomes of complex interventions but to extend this focus by identifying and defining the core mechanisms that contribute to the outcomes [40]. This establishes a descriptive model of how a program is expected to work in clinical practice, enhancing feasibility and acceptability within the scope of nursing practice. This aligns with a critical review, highlighting the importance of theory-building in the development of complex nursing interventions [41]. Given the scarcity of available theories to guide interventions in clinical nursing practice, researchers may be inclined to draw upon theories and conceptual frameworks from other disciplines [41]. This is exemplified well in our development of REDUCE as a nurse-led intervention and accounts for the need to provide detailed information about the role of theory in the conceptualization and development of the intervention. Detailing the underlying theoretical mechanisms, this study sought to build into REDUCE; this allows future studies, including metasyntheses and realist reviews, to establish a deeper understanding of how and why, for whom, and under what circumstances the program works [41].

### Mandatory Core Intervention Elements Allow Flexibility in Intervention Elements

A newly published systematic review by Diribe et al [42] of psychological interventions for adults with T1D suggests that psychological interventions, provided by nonpsychologists, incorporating self-determination theory produce effects equal to those delivered by qualified psychological therapists. These interventions build on cognitive behavioral theory, although it is claimed that such studies do not always control for confounding factors [42]. This finding provides support for the premises upon which REDUCE was developed, given that it is provided by nurses due to limited access to psychologists in diabetes care, and includes professional skill set development that aligns with the framework of self-determination theory [32]. The review by Diribe et al [42] also found that a more individualized approach, which considers patient profile and treatment of T1D, seems to be effective—a principle well-matched with REDUCE, as it is a tailored intervention targeting the 7 most common sources of diabetes distress specific to group participants. REDUCE contains only a few core intervention elements that are mandatory to use, allowing DSN and adults with T1D much flexibility in the selection and activation of relevant intervention elements. This practice aligns with the newly updated MRC framework, emphasizing that standardization should be related to the underlying key processes and functions rather than to the specific form of components [23,43]. In line with this, the MRC framework advocates for a theoretical deconstruction into components and an agreement about degree of flexibility in the delivery [23]. This approach allows the implementation of a complex intervention to vary across different contexts while maintaining the mechanisms and functions.

Fisher et al [43] used the concept of *equifinality* to clarify how different intervention approaches that are based on diverse theoretical concepts can be effective in fulfilling similar key functions to achieve similar goals. Thus, the use of a variety of interventions may lead to programs offering a broader range of approaches with greater appeal to adults with T1D and diabetes distress. In line with this, the REDUCE program has incorporated a variety of methods based on diverse approaches to offer a broad range of tools with variations of appeal for enhancing emotion regulation and peer-support, aiming to reduce diabetes distress.

### Future Directions of the Study

Following the updated MRC framework, future studies need to clarify specific conditions and contextual factors underpinning the mechanism of change [23]. To supplement the program theory of REDUCE, attention should, therefore, extend beyond the design of REDUCE and the underlying theoretical mechanisms. A focus on contextual conditions is needed to comprehend the mechanisms of change and the resources required for an effective intervention that is likely to be implementable, sustainable, and scalable in real-world practice. Improved program theory, including the identification of contextual factors, will also help inform the transferability of REDUCE across settings and help decision makers with valuable evidence and understanding [23].

On the basis of the findings from the face-to-face study, REDUCE is currently being adapted to a web-based format. Adaptation to a web-based format involves extensive patient and public involvement, with ongoing input from users and relevant stakeholders such as people with diabetes, diabetes specialists, web designers, and experts in web-based facilitation. Their contribution will ensure that the web-based format and content will be appropriately adapted to be useful in real-world settings and relevant to the target population. Nevertheless, the current face-to-face version of REDUCE builds primarily on components from the US diabetes distress reduction intervention T1-REDEEM [19], mainly consisting of web-based video meetings with adults with T1D and group facilitators. Therefore, we expect that the face-to-face format of REDUCE is easily adaptable to a web-based format.

However, adapting REDUCE to a web-based format requires that participants can access the visual material. Then, a key advantage of a web-based format will be that adults with T1D can reflect and read the visual material at their own time and pace over a long distance. In addition, a review of online group–based patient education interventions [44] highlights that group facilitator training and guidelines for web-based group sessions often receive limited attention [44]. Thus, the review emphasizes the importance of technological and educational skills to promote group cohesion and peer support online, which is a crucial mediator for interventional effect [44] and should be prioritized in future research when adapting to a web-based format. Furthermore, it is well known that social support and emotion-regulation approaches are crucial mechanisms for reducing diabetes distress [19,37]. Thus, peer support is a core mechanism in the current face-to-face version of REDUCE. While adapting REDUCE for web-based delivery, it will be relevant to explore and be inspired by existing digital sources such as social media forums and other relevant web-based chat communities that promote peer-support. However, while social media offers opportunities for peer support, there is an increasing attention on both the potential benefits and limitations in the context of diabetes care. Research has shown that social media can contribute to the spread of misinformation, which may negatively interfere with the daily diabetes management of individuals by exposing them to inaccurate or even harmful information [45,46]. However, this was mainly raised by health care professionals who were particularly skeptical and emphasized these risks, expressing concerns that misleading information could lead to unrealistic expectations and potentially dangerous actions among people with diabetes [47]. Nevertheless, for some adults with diabetes, social media offers a comfortable space to discuss sensitive topics more openly [48]. In addition, a review by Oser et al [49] suggests that participation in diabetes-specific online communities is primarily motivated by the need for social support in adults with diabetes, which is often perceived as inadequately provided by clinicians.

Moreover, REDUCE is currently being adapted for adults with type 2 diabetes. Thus, a substantial portion of the content form the original REDUCE program, designed for adults with T1D, has been directly transferred to this new version However, REDUCE consists of visual conversation tools specifically addressing the unique sources of diabetes distress for adults with T1D. Common stressors and worries in T1D, such as technology challenges, management difficulties, and hypoglycemia distress, differ from those typically seen in type 2 diabetes. Thus, the type 2 intervention is recommended to contain the most common concerns and worries for adults with type 2 diabetes. The adaptation process ensures that the core elements of the intervention remain applicable, while adjustments are made to address the specific needs and challenges faced by adults with type 2 diabetes.

### Strengths and Limitations of the Study

Due to the COVID-19 pandemic, the recruitment method primarily included adults with T1D through a diabetes panel affiliated with both Steno Diabetes Center Copenhagen and Steno Diabetes Center Zealand. This may have inadvertently introduced selection bias, raising concerns about the degree of transferability. Consequently, a limitation was that the participants who contributed to the adaptation of the intervention were primarily older than 40 years, predominantly female, and well-educated. However, diabetes distress can occur at any age and stage of living with T1D, although it is more common among women, younger individuals, those newly diagnosed, or those with complications [50]. In addition, the intensity of diabetes distress can fluctuate and often worsen during challenging situations due to the following reasons: (1) unexplained blood sugar fluctuations, (2) issues with diabetes technology, (3) pressure of managing diabetes perfectly, (4) changes in treatment plans, (5) onset or worsening of complications, or (6) stressful life events or changes in routine [51]. This highlights the need for ongoing support in life with T1D. Yet, the onset of T1D can occur at any age; recent epidemiological data have shown that the incidence of T1D increases with age and more than half of all new cases of T1D occur in adulthood [52,53]. Importantly, REDUCE does not target adults with new onset T1D as other care priorities have been identified in an evidence-based study for this target group [54]. A strength of the study was that a characteristic of adults with T1D participating in the study was diversity in the sample, including participants with both adult- and childhood-onset T1D. Another strength of the study was that the prototypes of the methods were pilot tested in groups with much more variety in those characteristics [37]. Further research involving a more diverse sample of adults with T1D is essential for broader applicability. Furthermore, a mixed methods approach to document relevant proximal outcomes of the design process would have been helpful for increased comprehension, acceptability on the part of patients, or feelings of competence on the part of the clinicians. These aspects should be explored in future studies.

### Conclusions

While various international diabetes guidelines highlight the significance of implementing interventions in clinical practice to reduce diabetes distress among adults with diabetes, such interventions are rarely prioritized as part of routine diabetes care. In this study, we refined or made changes to a preliminary set of prototypes on the basis of existing programs and methods by integrating the perspectives of adults with T1D, clinical health care professionals, and relevant experts within the field to further design, develop, and adjust conversation tools and a training manual to reduce diabetes distress. These tools can serve as valuable aids in assisting specialized diabetes nurses for supporting adults with T1D in reducing moderate to severe levels of diabetes distress. It will be important to conduct more studies to assess the long-term effectiveness of this nurse-led, diabetes-reduction intervention that focuses on psychosocial well-being and glycemic outcomes.

## Appendix

Multimedia Appendix 1 Overview of all visual conversation tools.

## Abbreviations

DSN: diabetes specialist nurse
MRC: Medical Research Council
REDUCE: Evidence-Based Nurse-Led Intervention to Reduce Diabetes Distress Among Adults With Type 1 Diabetes and Diabetes Distress
T1D: type 1 diabetes
T1-REDEEM: Randomized Controlled Trial to Reduce Diabetes Distress Among Adults With Type 1 Diabetes

## Data Availability

The datasets analyzed during this study are not publicly available because they contain information that could identify specific participants, but they are available from the corresponding author on reasonable request.
