# Supplementary material for: An Evidence-Based Nurse-Led Intervention to Reduce Diabetes Distress Among Adults With Type 1 Diabetes and Diabetes Distress (REDUCE): Development of a Complex Intervention Using Qualitative Methods Informed by the Medical Research Council Framework
Source: JMIR Form Res. 2024 Oct 18;8:e58658. doi: 10.2196/58658 (PMC11530732; doi:10.2196/58658)
Supplement: Multimedia Appendix 1 [file formative_v8i1e58658_app1.docx]

**Multimedia Appendix 1.** Overview of all visual conversation tools.

| **Tool and aim** | | **Content and strategies** | **Illustration** | **Mechanisms** |
| --- | --- | --- | --- | --- |
| Arrival and welcoming^a^: to establish a safe and trusting, nonjudgmental group environment | | - To outline the rules for group interactions - To providing information about the overall framework of the meetings and practical details - To facilitate a brief introduction of participants, enabling the expression of their expectations, preferences, and needs | 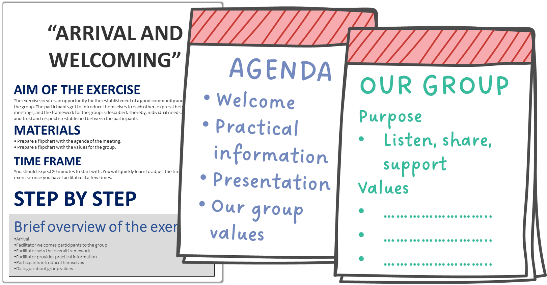 | - To establish confidentiality and a respectful group atmosphere - To kick-start active listening and open communication for sharing - To create a clear structure and set boundaries for maintaining privacy to guide supportive group interactions - To enhance empathy and group cohesion |
| Key points of the meeting^a^: to summarize group meetings by facilitating take-home messages from the group meeting | | - To create a sheet with questions to prompt reflection and articulation of important takeaways from the meeting | 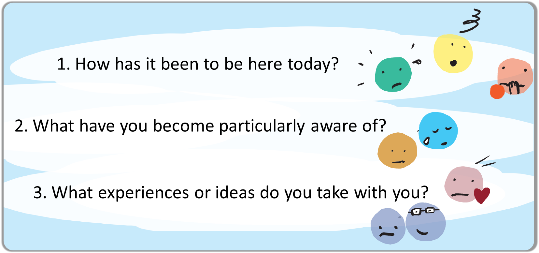 | - To provide a sense of closure and the transition from the group session to everyday life - To help participants reflect on the main insights or new strategies to manage the diabetes distress they have acquired from the meetings |
| Arrival and recap of the previous session^a^: to provide transparency and establish continuity by revisiting topics from the last meeting | | - To outline the content of today’s meeting - To summarize rules for group interactions, if needed - To facilitate a brief check-in and reflection on the interim period since the previous meeting | 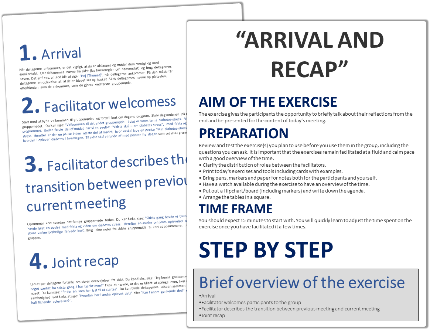 | - To establish confidentiality and a respectful group atmosphere - To promote active listening and open communication for sharing - To create a clear structure and set boundaries for maintaining the privacy that guides supportive group interactions - To enhance empathy and group cohesion |
| My diabetes story^a^: participants share and listen to other participants’ diabetes stories | | - To create a single visual conversation tool with open-ended questions for prompting reflections on different life events during the course with diabetes | 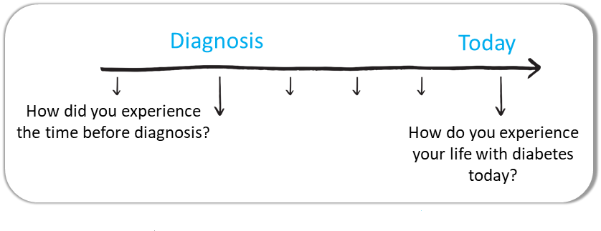 | - Icebreaker gradually open up emotions and prompt peer support and is a positive influence on participants’ willingness to share experiences - To provide empathy that promotes a broader understanding of diverse experiences and encourages a more inclusive and tolerant group dynamic |
| Did you know?—about diabetes distress^a^: to provide knowledge and improve understanding of diabetes distress | | - To create 13 cards with facts and knowledge about diabetes distress, its prevalence, causes, symptoms, responses, and potential consequences in a way that is person-centered, accessible, and understandable | 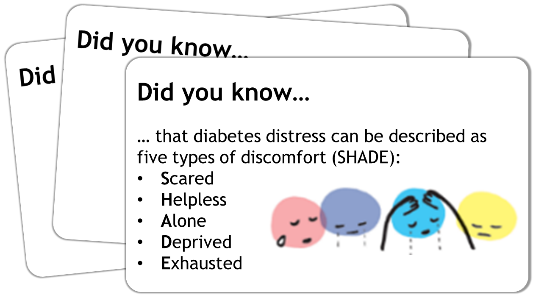 | - To improve the understanding of diabetes distress, create self-awareness, and reduce misconceptions about diabetes distress - To increase awareness of thoughts, emotions, and behaviors and a capability to recognize emotional patterns - To provide information on diabetes distress to legitimize, acknowledge, and normalize common emotional worries, which can reduce isolation and self-blame associated with diabetes distress |
| My diabetes distress^a^: to identify individual sources of diabetes distress and discuss how it affects everyday life | | - To use a set of 31 cards containing illustrations and statements capturing common worries and frustrations of individuals living with diabetes - To sort the statements made by participants into 3 distinct piles: *agree*, *maybe*, and *disagree* - To provide 1 visual illustration describing the unique sources of diabetes distress covered by the Type 1 Diabetes Distress Scale | 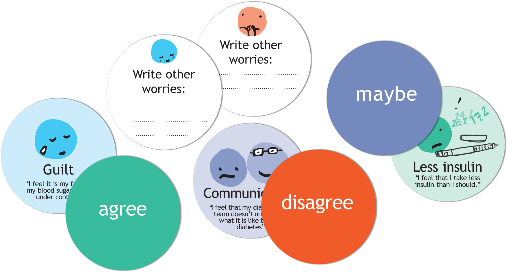 | - To support the recognition of common patterns by identifying unique sources of diabetes distress, which can help participants gain greater self-awareness, reframe self-perception, and increase clarity |
| **Powerlessness** | | | | |
|  | Worries about the future: to discuss worries about diabetes complications, see worries from a new perspective, and reflect on strategies to manage fears of diabetes complications | - To create 1 exercise sheet with 2 questions to be answered on a scale from 0 to 10 - To provide 4 cards titled *Did you know about the complications associated with diabetes distress?*—including facts about the actual risk of serious complications for adults with type 1 diabetes - To provide 1 question sheet titled *Diabetes in the Future* with open-ended questions to promote emotion- regulation skills | 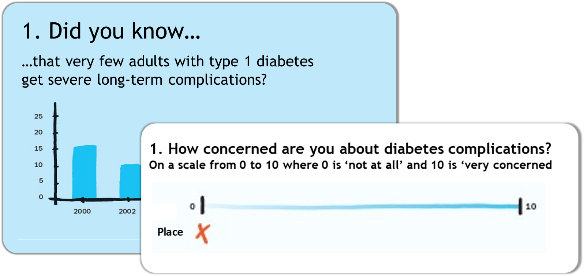 | - To provide evidence-based knowledge about the risk of diabetes complications, which can reframe unrealistic thoughts about the actual risk, encouraging a more positive, balanced, and rational perspective on illness perceptions - To prompt participants to contemplate the essence of what it means to be in glycemic target - To gauge the intensity of concerns, support self-awareness, and overtly label the extent of worries - To help communicate feelings more accurately and track levels of concern over time |
|  | Management of diabetes worries: to enable participants to identify, observe, and manage worries associated with diabetes | - To use a single exercise sheet consisting of two circles on a sheet, with the inner circle delineating worries within one’s control, while the outer circle highlighting worries beyond one’s control (Participants are asked to categorize various diabetes concerns into *My Control* and *Beyond My Contro*l categories to direct attention on efforts that are influenceable) | 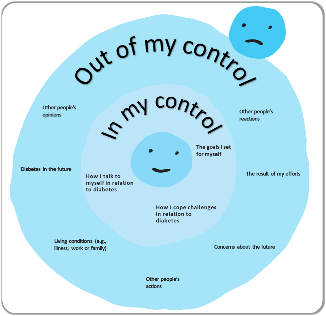 | - To support participants in identifying patterns on the amount of time and energy spent on worries, actions, and reactions they have control over, rather than getting stuck on uncontrollable concerns - To mentally establish nonreactivity to unnecessary worries that are beyond their control and redirect the focus on worries within their control for seeking solutions - Facilitating the development of strategies to redirect attention to a meaningful focus instead of dwelling on the past or worrying about the future can eliminate rumination, gain perspective, practice nonreactivity to emotions, and formulate proactive approaches to address concerns when they arise |
| **Management distress** | | | | |
|  | Worries about my blood glucose: to address the impact of blood sugar levels on daily life and facilitate discussions of strategies to overcome worries | - To create 1 sheet with statements capturing the daily frustrations associated with blood glucose fluctuations and another sheet with reflective questions designed to inspire new ideas to manage frustrations of individuals experiencing blood glucose fluctuations in everyday life | 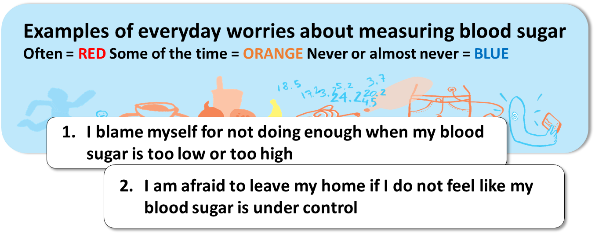 | - To improve understanding of worries related to measuring blood glucose, contributing to a sense of validation, clarity, and self-awareness |
| **Hypoglycemia distress** | | | | |
|  | Worries about low blood glucose: to address how concerns about low blood glucose impact everyday life and explore strategies for managing worries | - To provide a set of 23 cards containing illustrations and statements capturing various concerns about low blood glucose   and to sort participant statements into 3 distinct piles: *agree*, *maybe*, and *disagree*   - To include questions that prompt peer discussions about new ideas to manage concerns | 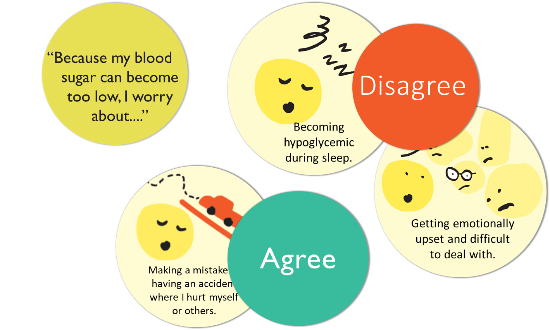 | - To improve the understanding of challenging emotions related to low blood glucose, which contributes to a sense of validation, clarity, and reduction in shame and blame |
| **Friends and family distress and negative social perceptions** | | | |  |
|  | Friends and family support: to discuss frustrations about social relations and develop strategies for managing social challenges | - To provide a set of 24 cards with illustrations and statements capturing the distress of friends and family as well as frustrations related to negative social perceptions about diabetes and to sort the statement into 3 distinct piles: *agree*, *maybe*, and *disagree* - To provide 5 cards titled *Did you know*?—with information on social relations with facts about the impact of social relations and stigma in adults with type 1 diabetes - To create 1 question sheet with open-ended questions for promoting emotion- regulation skills | 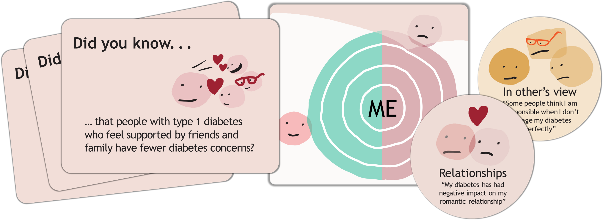 | - To improve the understanding of challenges related to social support and negative judgments from others about diabetes being common and normal, contributing to a sense of validation - To motivate participants to clarify resources that can provide social support, which can alleviate feelings of isolation - To provide questions for encouraging participants to reflect on the emotional support they receive and need from their social relationships - To foster open discussions of stigma experiences linked to diabetes, which can destigmatize and alleviate feelings of shame, guilt, and isolation - To reduce self-stigma, which can enhance self-perception and greater self-compassion and improve overall quality of life |
| **Eating distress** | | | | |
|  | Food and eating: to address how food and eating impact everyday life with diabetes and explore strategies for managing worries | - To use a set of 20 cards with illustrations and statements capturing common frustrations about food and eating when living with diabetes - To sort the statements of the participants into 3 distinct piles: *agree*, *maybe*, and *disagree*   (Questions are included to prompt peer discussions about new ideas to manage concerns.) | 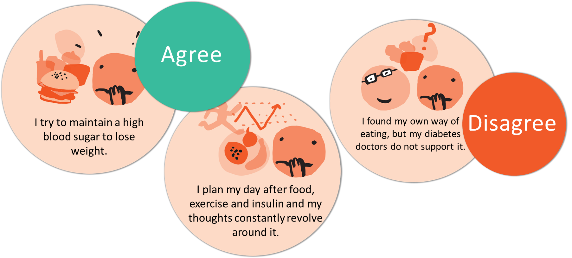 | - To improve understanding of challenging emotions related to low blood glucose, contributing to a sense of validation, clarity, and reduce shame and blame |
| **Physician distress** | | | | |
|  | Support from diabetes providers: to address frustrations regarding diabetes providers and develop strategies to improve communication in consultations | - To provide a single exercise sheet to address and share specific issues related to physician distress and to explore potential constructive solutions in the communication process with the diabetes provider | 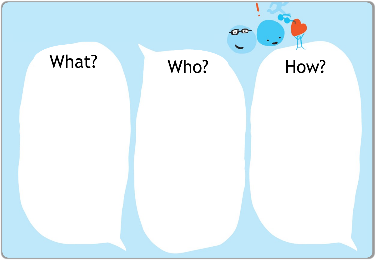 | - To identify and develop strategies to enhance communication with diabetes providers   (This involves exploring effective communication techniques, setting clear expectations, and expressing needs and concerns assertively)   - To express concerns, ask questions, and actively participate in decision-making processes related to diabetes care |
| **Gains from the group meeting and management of diabetes distress in the future** | | | | |
|  | Final recap of group meetings^a^: to reflect on gains from all the group sessions and plan strategies to manage diabetes distress in the future | - To provide a single exercise sheet to support reflection on gains from group sessions and plan strategies to manage diabetes distress in the future | 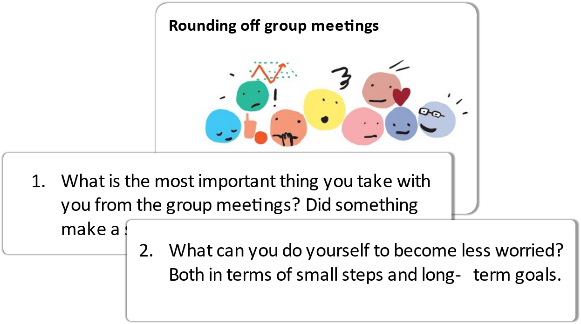 | - To summarize key learnings, achievements, and changes experienced during the group meetings - To recognize and acknowledge personal progress in managing diabetes distress - To take an active role in participants’ emotional well-being by reflecting on gains and planning for the future, thereby empowering participants - To practice self-compassion by reflecting on kindness as a strategy to reduce diabetes distress |

^a^These worksheets are mandatory.
